# Association of Cytotoxic T-Lymphocyte-Associated Protein 4 (*CTLA4*) Gene Polymorphisms with Autoimmune Thyroid Disease in Children and Adults: Case-Control Study

**DOI:** 10.1371/journal.pone.0154394

**Published:** 2016-04-25

**Authors:** Wei-Hsin Ting, Ming-Nan Chien, Fu-Sung Lo, Chao-Hung Wang, Chi-Yu Huang, Chiung-Ling Lin, Wen-Shan Lin, Tzu-Yang Chang, Horng-Woei Yang, Wei-Fang Chen, Ya-Ping Lien, Bi-Wen Cheng, Chao-Hsu Lin, Chia-Ching Chen, Yi-Lei Wu, Chen-Mei Hung, Hsin-Jung Li, Chon-In Chan, Yann-Jinn Lee

**Affiliations:** 1 Department of Pediatrics, MacKay Children’s Hospital, Taipei, Taiwan; 2 Department of Endocrinology and Metabolism, MacKay Memorial Hospital, Taipei, Taiwan; 3 Department of Medicine, MacKay Medical College, New Taipei City, Taiwan; 4 Institute of Mechatronic Engineering, National Taipei University of Technology, Taipei, Taiwan; 5 MacKay Junior College of Medicine, Nursing and Management, New Taipei City, Taiwan; 6 Department of Pediatrics, Chang Gung Memorial Hospital, Tao-Yuan, Taiwan; 7 College of Medicine, Chang Gung University, Tao-Yuan, Taiwan; 8 Department of Medical Research, MacKay Memorial Hospital Tamsui, New Taipei City, Taiwan; 9 Department of Pediatrics, MacKay Memorial Hospital HsinChu, Hsin-Chu, Taiwan; 10 Department of Pediatrics, Chiayi Christian Hospital, Chia-Yi, Taiwan; 11 Department of Pediatrics, Changhua Christian Hospital, Chang-Hua, Taiwan; 12 Department of Pediatrics, Hsinchu Cathay General Hospital, Hsin-Chu, Taiwan; 13 Department of Pediatrics, St. Martin De Porres Hospital, Chia-Yi, Taiwan; 14 Department of Pediatrics, School of Medicine, College of Medicine, Taipei Medical University, Taipei, Taiwan; 15 Institute of Biomedical Sciences, MacKay Medical College, New Taipei City, Taiwan; Baylor College of Medicine, UNITED STATES

## Abstract

Autoimmune thyroid disease (AITD), including Graves disease (GD) and Hashimoto disease (HD), is an organ-specific autoimmune disease with a strong genetic component. Although the cytotoxic T-lymphocyte-associated protein 4 (*CTLA4*) polymorphism has been reported to be associated with AITD in adults, few studies have focused on children. The aim of our study was to investigate whether the *CTLA4* polymorphisms, including -318C/T (rs5742909), +49A/G (rs231775), and CT60 (rs3087243), were associated with GD and HD in Han Chinese adults and children. We studied 289 adult GD, 265 pediatric GD, 229 pediatric HD patients, and 1058 healthy controls and then compared genotype, allele, carrier, and haplotype frequencies between patients and controls. We found that *CTLA4* SNPs +49A/G and CT60 were associated with GD in adults and children. Allele G of +49A/G was significantly associated with GD in adults (odds ratio [OR], 1.50; 95% confidence interval [CI], 1.21–1.84; corrected *P* value [*Pc*] < 0.001) and children (OR, 1.42; 95% CI, 1.15–1.77; *Pc* = 0.002). Allele G of CT60 also significantly increased risk of GD in adults (OR, 1.63; 95% CI, 1.27–2.09; *Pc* < 0.001) and GD in children (OR, 1.58; 95% CI, 1.22–2.04; *Pc* < 0.001). Significant linkage disequilibrium was found between +49A/G and CT60 in GD and control subjects (D’ = 0.92). Our results showed that *CTLA4* was associated with both GD and HD and played an equivalent role in both adult and pediatric GD in Han Chinese population.

## Introduction

Autoimmune thyroid disease (AITD), including Graves disease (GD) and Hashimoto disease (HD), is an organ-specific autoimmune disease characterized by the presence of autoantibodies and T cell-mediated autoimmunity against self-antigens [1]. Both GD and HD involve similar genetic background and additional environmental and hormonal factors. Antibody-mediated thyroid stimulation prominently occurs in GD, whereas lymphocyte- and cytokine-mediated thyroid apoptosis predominates in HD, but overlap may occur [1]. They are the most prevalent autoimmune endocrinological diseases in children and adolescents [2], and are estimated to affect approximately 1% of the general population [3]. Although the exact etiology has not been fully clarified, the current hypothesis is that a complex interplay between genetic and environmental factors causes AITD [4–6].

AITD has been found to be clustered in families [7]. The risk ratio for a female sibling (λs) of a proband with GD is 15–20 [8]. The concordance rate of GD is 20–35% in monozygotic twins, but only 3–7% in dizygotic twins [9]. Twin studies also reveal that genetic factors contribute to about 75% of the development of AITD [10]. These observations strongly suggest that genetic factors are important in the pathogenesis of AITD.

The most important gene involved in AITD is the *HLA-DR* locus [4]. Other identified candidate genes that confer susceptibility to AITD can be classified into the following two groups: (1) immune regulatory genes: cytotoxic T-lymphocyte-associated protein 4 (*CTLA4*); protein tyrosine phosphatase, non-receptor 22 (*PTPN22*); interleukin 2 receptor (*IL-2R*); and (2) thyroid-specific genes: the thyroglobulin gene (*TG*) and the thyroid stimulating hormone receptor gene (*TSHR*) [4].

The *CTLA4* gene encodes a transmembrane regulatory protein, cytotoxic T-lymphocyte-associated protein 4, which is expressed on activated T cells and negatively regulates their function [11]. *CTLA4* competes with CD 28 binding with its ligand B7 on antigen presenting cells [12], raises the threshold of T cell activation [13], increases T cell motility and overrides T cell receptor induced stop signal required for stable conjugate formation between T cells and antigen presenting cells [14]. Several polymorphic sites in the gene, including C>T polymorphism in the promoter –318 (rs5742909) [15], A>G polymorphism in exon 1 +49A/G (rs231775) [16], microsatellite (AT)_n_ repeat in the 3’-untranslated region (UTR) [17], and three single nucleotide polymorphisms (SNPs) in the 6.1-kb 3’ noncoding region, CT60 (rs3087243G>A), JO31 and JO30 [18, 19], are associated with organ-specific autoimmune disorders in several racial groups [18–25]. Among them, +49A/G and CT60 are the most widely investigated markers of autoimmune diseases [26]. These two polymorphisms are associated with thyroid antibody production [27, 28], GD relapse [25], Graves ophthalmopathy [29, 30], and susceptibility to GD [23, 31] and HD [32, 33]. Recently, many meta-analyses have also demonstrated the association between *CTLA4* polymorphisms and AITD [34–38]. However, studies on GD in pediatric populations are limited and inconsistent [39–41]. Whether *CTLA4* polymorphisms deliver different risks to adult and pediatric GD patients needs to be clarified [42, 43].

We hypothesized that the association between the *CTLA4* gene and GD might be different between children and adults. Therefore we aimed to investigated *CTLA4* polymorphisms (-318C/T, +49A/G, and CT60) in pediatric and adult GD patients and controls of Han Chinese ethnicity. In addition, we also analyzed these polymorphisms in HD children with the hope that the results on HD might provide more clues to help us solve the issue. The genotype, allele, carrier, and haplotype frequencies of each disease group were compared with those of ethnically matched controls.

## Material and Methods

### Patients

#### Graves disease

The subjects were 554 unrelated patients consisting of 289 adults from endocrine clinics and 265 children from pediatric clinics. An adult was defined as a person who was 18 years of age or older at diagnosis for patients or at blood sampling for controls. The adult patients were 47 men and 242 women. Their mean age at diagnosis was 33.6 years (SD = 10.2, range 18.2–66.7 years). The pediatric patients were 43 boys and 222 girls. Their mean age at diagnosis was 10.7 years (SD = 3.4, range 2.7–17.9 years). GD was diagnosed on the basis of clinical and laboratory evidence, including thyrotoxicosis, diffuse goiter, with or without ophthalmopathy, elevated free T4/total T4 levels, suppressed TSH levels, and presence of autoantibodies to TSH receptor and thyroglobulin, microsomes, or both [44, 45].

#### Hashimoto disease

The subjects were 229 unrelated patients from pediatric clinics, including 65 boys and 164 girls. Their mean age at diagnosis was 10.8 years (SD = 3.6, range 2.5–17.8 years). HD was diagnosed on the basis of clinical and laboratory evidence including hypothyroidism, diffuse goiter, depressed free T4/total T4 levels, elevated TSH levels, and presence of autoantibodies to thyroglobulin, microsomes or TSH receptor [46, 47].

#### Controls

The 1058 control subjects consisted of 478 males and 580 females. They included hospital personnel and individuals who underwent routine health examinations or minor surgery. None of them had a history of autoimmune disease. Ages were recorded for 575 subjects (mean ± SD = 15.6 ± 9.9, range 5.2–65.1 years), but the remaining 483 were only noted to be adults. All patients and controls were Han Chinese in Taiwan. The institutional review board of MacKay Memorial Hospital approved this study and all subjects and/or their guardians provided written informed consent.

### Selection of single nucleotide polymorphisms (SNPs)

dbSNP -318C/T (rs5742909C>T) is at position -318 of the promoter region of the *CTLA4* gene, +49A/G (rs231775A>G) is at position +49 in exon 1, and CT60 (rs3087243G>A) at position +6230 in the 3' untranslated region [18]. Individuals with the -318 T allele or +49 A/A genotype have higher *CTLA4* expression [48]. The genotype G/G of CT60 is associated with a 50% decrease in the soluble *CTLA4* isoform [18].

#### DNA extraction

Genomic DNA was extracted from fresh or frozen peripheral blood leukocytes by standard techniques.

#### Genotyping of the *CTLA4* gene

The -318C/T, +49A/G, and CT60 polymorphisms were typed by using polymerase chain reaction–restriction fragment length polymorphism (PCR-RFLP) in 30% of the patients and 82% of controls. The procedure is detailed in our previous report [49]. The remaining patients and controls were typed using the Pre-Developed TaqMan Allelic Discrimination Assay (Applied Biosystems, Foster City, CA, USA). Briefly, polymerase chain reactions (PCR) were performed using TaqMan assay plates. Reactions contained 10 ng genomic DNA, 5 μl TaqMan Universal PCR Master Mix, 0.25 μl 40× Assay Mix, and ddH_2_O to a final volume of 10 μl. Thermal cycle conditions were as follows: denaturation at 95ºC for 10 min, followed by 40 cycles of denaturation at 92ºC for 15 sec, and annealing and extension at 60ºC for 1 min. Reactions were performed and read on a 7500 Fast Real-Time PCR System (Applied Biosystems). The allelic specific fluorescence data from each plate were analyzed by using the SDS v1.4.0 software (Applied Biosystems) to automatically determine the genotype of each sample.

### Statistical power

We designed the study to have a power of >90% at a 5% significance level to determine a genotype relative risk of 1.61 for +49A/G (rs231775) G/G of *CTLA4* in a recessive mode with a prevalence of 0.5% in GD [2] according to the Genetic Power Calculator [50]. We would need to study 223 cases and 1115 controls to be able to reject the null hypothesis.

### Statistical analysis

We assessed the Hardy-Weinberg equilibrium for the SNPs, estimated the frequencies of haplotypes with an accelerated expectation-maximization algorithm, and tested pairwise linkage disequilibrium (LD) between the SNPs in both patients and controls using Haploview 4.2 [51].

Difference in genotype, allele, carrier, and haplotype distributions between patients and controls were assessed by using the χ^2^ test. Odds ratios (OR) and 95% confidence intervals (CI) were also calculated [52].

The Bonferroni correction, *Pc* = 1 –(1-*P*)^n^, was used for multiple comparisons where *Pc* is the corrected *P* value, *P* the uncorrected value, and n the number of comparisons [53]. In this study, n is 2 for each genotype, allele, or carrier (for simultaneously testing genotype, allele, and carrier frequencies [54]) but no correction for testing the three SNPs because of significant linkage between them and 4 for each of the four haplotypes [55]. A *Pc* value of less than 0.05 was considered statistically significant [56].

To test the influence of LD and detect a stronger association between alleles at two adjacent loci, we tested association with one locus in the presence or absence of the associated allele at the second locus [53].

## Results

All patients and controls were successfully typed. We randomly selected and genotyped 30 specimens using both PCR-RFLP and TaqMan Allelic Discrimination Assay and found that the results were consistent. We detected no significant difference in the genotype, allele and carrier frequencies between pediatric and adult controls (data not shown). Therefore we pooled both control groups together for data presentation and analysis.

**Fig 1:**
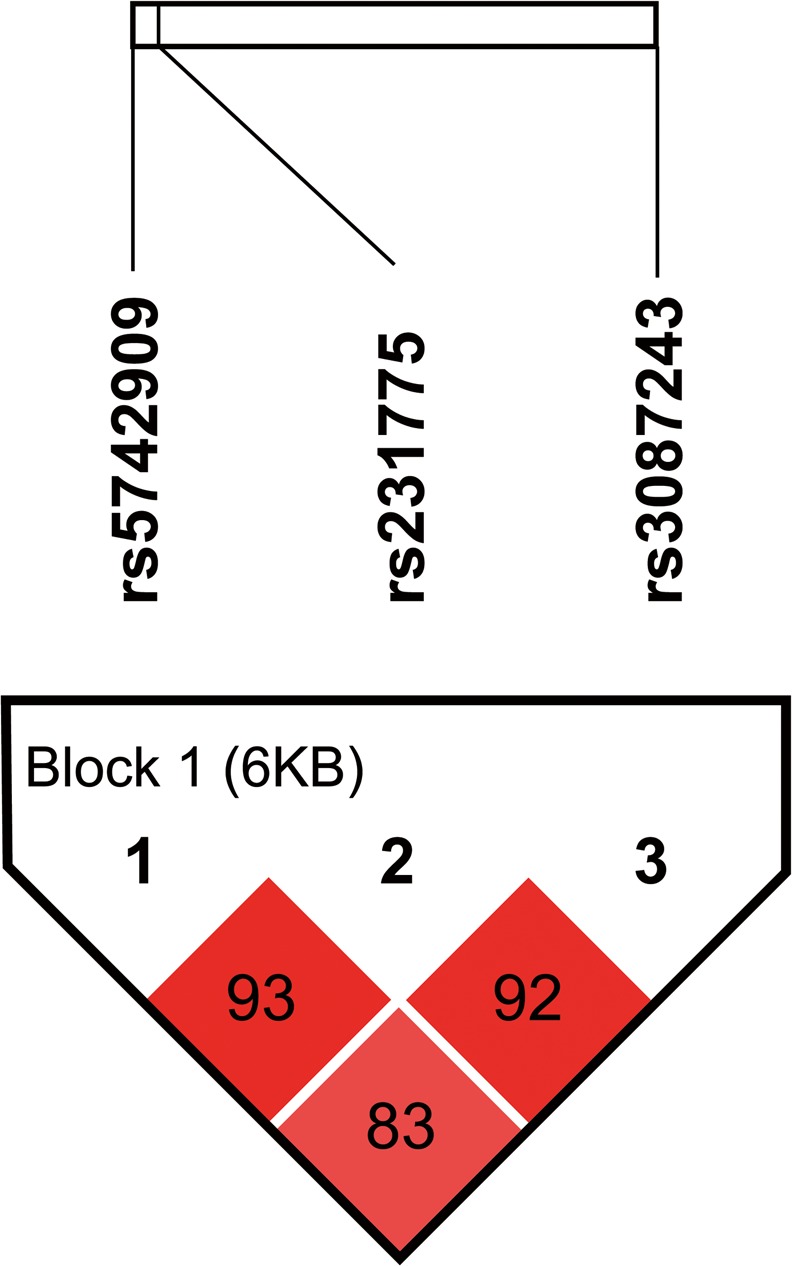
Linkage disequilibrium (LD) plot in 1058 controls. The ticks on the uppermost white bar denote the genotyped SNPs and their relative sites. The SNP pairwise information is plotted as boxes between these ticks. The number in the box denotes the D' value between each pair of SNPs. The intensity of the box color is proportional to the strength of the LD. The block is generated by solid spine of LD method in Haploview 4.2.

The genotype distributions of SNPs -318C/T, +49A/G, and CT60 in patients and controls were in Hardy-Weinberg equilibrium (*P =* 0.26–0.98) (Tables 1–3). The three SNPs were in LD with each other in the controls (D' ≥ 0.83) (Fig 1). The LD between them is strong (D' > 0.8) [57] and the three SNPs were in one block by solid spine of LD of Haploview 4.2 [51].

**Table 1:** Polymorphism of -318 C/T (rs5742909) of the *CTLA4* gene in adult Graves disease, pediatric Graves disease, Hashimoto disease patients and controls.

	Adult Graves disease		Pediatric Graves disease		Pediatric Hashimoto disease		Controls
	N = 289		N = 265		N = 229		N = 1058
Genotype	N (%)	OR (95%CI)	N (%)	OR (95%CI)	N (%)	OR (95%CI)	N(%)
C/C	232(80.3)	0.94(0.68–1.31)	215(81.1)	1.00(0.71–1.41)	185(80.8)	0.97(0.68–1.40)	859(81.2)
C/T	55(19.0)	1.10(0.79–1.54)	48(18.1)	1.04(0.73–1.47)	42(18.3)	1.05(0.73–1.52)	186(17.6)
T/T	2(0.7)	0.56(0.13–2.50)	2(0.8)	0.61(0.14–2.73)	2(0.9)	0.71(0.16–3.16)	13(1.2)
Allele							
C	519(89.8)	0.98(0.72–1.33)	478(90.2)	1.02(0.74–1.41)	412(90.0)	1.00(0.71–1.40)	1904(90.0)
T	59(10.2)	1.02(0.75–1.38)	52(9.8)	0.98(0.71–1.34)	46(10.0)	1.00(0.72–1.40)	212(10.0)
Carrier							
C	287(99.3)	1.79(0.40–7.96)	263(99.2)	1.64(0.37–7.29)	227(99.1)	1.41(0.32–6.30)	1045(98.8)
T	57(19.7)	1.06(0.76–1.47)	50(18.9)	1.00(0.71–1.42)	44(19.2)	1.03(0.71–1.48)	199(18.8)

Hardy-Weinberg test for adult GD: χ^2^ = 0.42, *P* = 0.81; pediatric GD: χ^2^ = 0.15, *P* = 0.93; pediatric HD: χ^2^ = 0.05, *P* = 0.97; controls: χ^2^ = 0.65

*P* = 0.72.

**Table 2:** Polymorphism of +49A/G (rs231775) of the *CTLA4* gene in adult Graves disease, pediatric Graves disease, Hashimoto disease patients and controls.

	Adult Graves disease		Pediatric Graves disease		Pediatric Hashimoto disease		Controls
	N = 289		N = 265		N = 229		N = 1058
Genotype	N(%)	OR(95%CI)	N(%)	OR(95%CI)	N(%)	OR(95%CI)	N(%)
A/A	15(5.2)	**0.46(0.27–0.81)**	19(7.2)	0.65(0.39–1.08)	15(6.6)	0.59(0.34–1.04)	112(10.6)
A/G	112(38.8)	0.79(0.61–1.04)	97(36.6)	**0.73(0.55–0.96)**	97(42.4)	0.92(0.69–1.23)	469(44.3)
G/G	162(56.1)	1.55*(1.20–2.02)	149(56.2)	1.56*(1.19–2.05)	117(51.1)	1.27(0.96–1.69)	477(45.1)
Allele							
A	142(24.6)	0.67**(0.54–0.83)	135(25.5)	0.70*(0.57–0.87)	127(27.7)	0.79(0.63–0.99)	693(32.8)
G	436(75.4)	1.50**(1.21–1.84)	395(74.5)	1.42*(1.15–1.77)	331(72.3)	1.27(1.02–1.59)	1423(67.2)
Carrier							
A	127(43.9)	0.64*(0.50–0.84)	116(43.8)	0.64*(0.49–0.84)	112(48.9)	0.79(0.59–1.05)	581(54.9)
G	274(94.8)	**2.16(1.24–3.77)**	246(92.8)	1.53(0.92–2.54)	214(93.4)	1.69(0.97–2.95)	946(89.4)

Significant values are in bold (*Pc* <0.05).

* *Pc* <0.01.

**Pc <0.001.

Hardy-Weinberg test for adult GD: χ^2^ = 0.60, *P* = 0.74; pediatric GD: χ^2^ = 0.34, *P* = 0.84; pediatric HD: χ^2^ = 0.74, *P* = 0.69; controls: χ^2^ = 0.04, *P* = 0.98.

**Table 3:** Polymorphism of CT60 (rs3087243) of the *CTLA4* gene in adult Graves disease, pediatric Graves disease, Hashimoto disease patients and controls.

	Adult Graves disease		Pediatric Graves disease		Pediatric Hashimoto disease		Controls
	N = 289		N = 265		N = 229		N = 1058
Genotype	N(%)	OR(95%CI)	N(%)	OR(95%CI)	N(%)	OR(95%CI)	N(%)
A/A	3(1.0)	**0.23(0.07–0.75)**	7(2.6)	0.60(0.27–1.34)	7(3.1)	0.69(0.31–1.56)	46 (4.3)
A/G	81(28.0)	**0.69(0.52–0.92)**	68(25.7)	0.61*(0.45–0.83)	67(29.3)	0.76(0.56–1.05)	382(36.1)
G/G	205(70.9)	1.66**(1.25–2.20)	190(71.7)	1.72**(1.28–2.31)	155(67.7)	**1.42(1.05–1.93)**	630(59.5)
Allele							
A	87(15.1)	0.61**(0.48–0.79)	82(15.5)	0.63**(0.49–0.82)	81(17.7)	0.74(0.57–0.97)	474(22.4)
G	49 (84.9)	1.63**(1.27–2.09)	448(84.5)	1.58**(1.22–2.04)	377(82.3)	1.34(1.04–1.74)	1642(77.6)
Carrier							
A	84(29.1)	0.60**(0.45–0.80)	75(28.3)	0.58**(0.43–0.78)	74(32.3)	**0.70(0.52–0.95)**	428(40.5)
G	286(99.0)	**4.33(1.34–14.04)**	258(97.4)	1.68(0.75–3.75)	222(96.9)	1.44(0.64–3.24)	1012(95.7)

Significant values are in bold (*Pc* <0.05).

* *Pc* <0.01.

**Pc <0.001.

Hardy-Weinberg test for adult GD: χ^2^ = 2.66, *P* = 0.26; pediatric GD: χ^2^ = 0.10, *P* = 0.95; pediatric HD: χ^2^ = 0.01, *P* = 1.0; controls: χ^2^ = 1.57, *P* = 0.46.

### -318C/T (rs5742909C>T)

The distributions of genotype, allele, and carrier of -318C/T were not significantly different between adult GD, pediatric GD, HD patients and controls (Table 1).

### +49A/G (rs231775A>G)

The SNP +49A/G was significantly associated with GD in adults and children, but only borderline significantly associated with HD in children (Table 2). Genotype G/G conferred a risk of 1.55 (95% CI, 1.20–2.02; *Pc* = 0.002) for GD in adults and of 1.56 (95% CI, 1.19–2.05; *Pc* = 0.002) in children. Allele G was significantly associated with GD in adults (OR, 1.50; 95% CI, 1.21–1.84; *Pc* < 0.001) and in children (OR, 1.42; 95% CI, 1.15–1.77; *Pc* = 0.002).

### CT60 (rs3087243G>A)

The SNP CT60 was also significantly associated with GD and HD (Table 3). Genotype G/G significantly increased the risk of GD in adults (OR, 1.66; 95% CI, 1.25–2.20; *Pc* < 0.001), GD in children (OR, 1.72; 95% CI, 1.28–2.31; *Pc* < 0.001) and HD in children (OR, 1.42; 95% CI, 1.05–1.93; *Pc* = 0.04). Allele G was significantly associated with GD in adults (OR, 1.63; 95% CI, 1.27–2.09; *Pc* < 0.001) and children (OR, 1.58; 95% CI, 1.22–2.04; *Pc* < 0.001).

### Haplotypes of the *CTLA* gene and Graves disease

Seven haplotypes were detected with two major haplotypes (CGG and CAA), which accounted for >84% in combined frequency in both patients and controls (Table 4). Haplotypes CGG and CAA were significantly associated with GD in adults and children, but the association with HD was not statistically significant in children. Haplotype CGG conferred a significant risk of GD in adults (OR, 1.29; 95% CI, 1.06–1.58; *Pc* = 0.05) and in children (OR, 1.51; 95% CI, 1.22–1.87; *Pc* < 0.001) while haplotype CAA was less frequently observed in adults and pediatric GD patients than in controls. The OR for GD was 0.58 (95% CI, 0.44–0.75; *Pc* < 0.001) in adults and 0.67 (95% CI, 0.51–0.86; *Pc* = 0.008) in children.

**Table 4:** Frequency of haplotypes of the *CTLA4* gene in adult Graves disease, pediatric Graves disease, Hashimoto disease patients and controls.

	Adult Graves disease		Pediatric Graves disease		Pediatric Hashimoto disease		Controls
	N = 578		N = 530		N = 458		N = 2116
Haplotype	N(%)	OR(95%CI)	N(%)	OR (95%CI)	N(%)	OR(95%CI)	N(%)
CGG	412(71.2)	1.29(1.06–1.58)	394(74.3)	1.51**(1.22–1.87)	326(71.2)	1.28(1.03–1.60)	1392(65.8)
CAA	77(13.3)	0.58**(0.44–0.75)	80(15.1)	0.67*(0.51–0.86)	77(16.8)	0.76(0.58–0.99)	446(21.1)
TAG	45(7.7)	0.81(0.58–1.14)	51(9.6)	1.03(0.74–1.42)	45 (9.8)	1.05(0.75–1.48)	199(9.4)
CAG	21(3.6)	1.86(1.09–3.17)	3(0.6)	0.28(0.09–0.91)	5(1.1)	0.55(0.21–1.39)	42(2.0)
Others	24(4.2)		2(0.4)		5(1.1)		36(1.7)

Significant values are in bold (*Pc* <0.05).

* *Pc* <0.01.

**Pc <0.001.

The order of SNPs in the haplotype is rs5742909-rs231775-rs3087243.

The haplotypes with a frequency of <2.0% in both patients and controls are grouped into "Others" and not compared.

### Detection of independent or stronger associations

We investigated whether carrier +49G or CT60G had an independent or stronger association with AITD using 2 by 2 comparison on +49A/G and CT60 between patients and controls [53]. There was significant LD between +49A/G and CT60 in the three groups of AITD patients and controls (S1 Table). We did not detect a significant independent or stronger association between carrier +49G or CT60G and AITD. Thus, a combination of +49A/G and CT60 polymorphisms was needed to enhance the role of the *CTLA4* gene in GD.

### Comparisons between subgroups stratified by gender

We compared genotype, allele, and carrier frequencies of +49A/G, CT60 between disease groups and controls stratified by gender. Association was statistically significant only in females. All trends of genetic association and effect sizes were similar in males and females (data not shown).

## Discussion

We demonstrated that SNPs +49A/G (rs231775) and CT60 (rs3087243) of the *CTLA4* gene were significantly associated with GD in both adults and children. Genotype G/G and allele G of +49A/G as well as genotype G/G and allele G of CT60 were significantly more frequent in adult GD and pediatric GD patients than in controls. Haplotype CGG conferred a significant risk of GD in adults and children.

Our case-control study did not demonstrate any significant association between the -318C/T polymorphism and AITD. One study focusing on this polymorphism and *CTLA4* protein expression showed that the -318T allele is associated with higher promoter activity and thus reduced autoimmunity [58]. However, most published studies do not show any association between -318 C/T and GD [59–62]. The homozygous T/T genotype is rare in our data set and in other populations [35, 61]. Therefore this SNP might not be important for susceptibility to AITD.

Our results suggested allele G of both +49A/G and CT60 were susceptibility factors for GD in adults and children. In addition, we found strong LD between +49A/G and CT60, but no stronger associations between +49A/G, CT60 polymorphisms and GD. This implied that the two SNPs were equally important genetic variants for GD in both children and adults. Likely, a single *CTLA4* variant is not solely causative and a haplotype consisting of several variants is responsible for the association with GD [63].

Carrier G of +49A/G confers a higher risk of GD susceptibility in children than in adults [43]. The strength of the association between +49A/G and GD is similar in Japanese children and adults [42]. Allele G of CT60 is associated with a 1.61-fold risk to develop GD in children [39], and a 1.89-fold risk to develop GD in adults in Taiwanese population [31]. Our results suggested that both +49A/G and CT60 rendered an equivalent risk of GD in children and adults. Collectively, *CTLA4* polymorphisms might play a similar role in conferring susceptibility to GD in both children and adults. Further large-scale studies are necessary to confirm this speculation.

The +49G allele encodes an alanine residue at codon 17 and is associated with inefficient glycosylation, decreased *CTLA4* cell surface expression [64], and reduced control of T cell proliferation [65, 66]. The CT60 polymorphism correlates with soluble *CTLA4* (s*CTLA4*, lacking exon 3) levels, with the disease-predisposing G allele rendering less s*CTLA4* mRNA [18]. This suggests that a combination of +49G and CT60G might confer a lesser *CTLA4* function, resulting in greater T-cell activity, stronger immune response, and a higher probability of autoimmunity. However, recently published data show a lack of correlation between the CT60 genotype and serum s*CTLA4* levels [67] or even higher serum s*CTLA4* levels in CT60G carriers [29]. GD patients have increased rather than decreased circulating s*CTLA4* levels [29, 68, 69]. The s*CTLA4* might inhibit T-cell activation by blocking the CD80/CD86-CD28 interaction in the earlier stage, but it might compete with membrane bound *CTLA4* for CD80/CD86, and prevent down-regulation of T cell response in the later stage [68]. Further functional studies are required to clarify the complex relationship between *CTLA4* polymorphisms, T-cell function and AITD.

In our study, only genotype G/G of CT60 was significantly associated with increased risk of pediatric HD. Although the remaining analyses on the allele and carrier distribution of CT60; on the genotype, allele, and carrier distribution of +49A/G; and on the haplotype distribution of *CTLA4* showed no significant association with HD, the direction of the effect was in accord with that in GD and suggested a possible similar association as in GD. The inability to reach statistical significance is probably due to the small sample size of HD patients. Bicek at al. reported that +49G and CT60G positively correlated with GD but not with HD [70]. In an updated meta-analysis, consistent associations between +49A/G [36], CT60 [71] and GD are detected in various ethnic populations, whereas associations between +49A/G [72], CT60 [71] and HD are only found in Asians, but not in Caucasians. Effect size of genetic factors, genetic backgrounds, gene-environment interactions, and multiple mechanisms of autoimmune disease are confounding factors [34, 73]. Our results supported the notion that *CTLA4* is important in the common pathogenic pathway leading to both GD and HD, but probably more involved in GD [70, 74].

Our study had several limitations. First, we did not include adult HD patients and were unable to compare the effects of *CTLA4* polymorphisms between pediatric and adult HD patients. Second, we did not conduct subgroup analysis stratified by levels of antithyroid antibodies, which might confound the results. Third, we could not distinguish whether the associated alleles were causative factors or just markers linked with true disease loci.

Our study showed a significant association between *CTLA4* polymorphism and GD in adults and children. The trend of the association between *CTLA4* and HD was similar with less effect size and less significance. Further studies are required to elucidate the complex relationship between *CTLA4* polymorphisms and AITD.

## Conclusion

*CTLA4* +49A/G and CT60 polymorphisms were associated GD in adults and children. Genotype G/G, allele G of +49A/G and genotype G/G, allele G of CT60 were found significantly more frequent in adults and children with GD than in controls. However, only genotype G/G of CT60 was significantly associated with HD. Our results showed that *CTLA4* was associated with both GD and HD, and played an equivalent role in both adult and pediatric GD in Han Chinese population.

## Supporting Information

S1 Table Two-by-two tests on carrier G of the +49A/G (rs231775) and carrier G of CT60 (rs3087243) between adult GD, pediatric GD, HD patients and controls. (DOCX)
