# Supplementary material for: Association of Cytotoxic T-Lymphocyte-Associated Protein 4 (CTLA4) Gene Polymorphisms with Autoimmune Thyroid Disease in Children and Adults: Case-Control Study
Source: PLoS One. 2016 Apr 25;11(4):e0154394. doi: 10.1371/journal.pone.0154394 (PMC4844099; doi:10.1371/journal.pone.0154394)
Supplement: S1 Table — (DOCX) [file pone.0154394.s001.docx]

S1a Table. Carrier distributions of the +49A/G (rs231775) and CT60 (rs3087243) of the *CTLA4* gene in adult GD, pediatric GD, HD patients and controls

| +49A/G | CT60 | Adult Graves disease | Pediatric Graves disease | Pediatric Hashimoto disease | Controls |
| --- | --- | --- | --- | --- | --- |
|  |  | N=289 | N=265 | N=229 | N=1058 |
| G | G | 274 | 246 | 214 | 944 |
| G | A | 0 | 0 | 0 | 2 |
| A | G | 12 | 12 | 8 | 68 |
| A | A | 3 | 7 | 7 | 44 |

S1b Table. Two-by-two tests on carrier G of the +49A/G (rs231775) and carrier G of CT60 (rs3087243) between adult GD, pediatric GD, HD patients and controls

|  | | Adult Graves disease | | Pediatric Graves disease | | Pediatric Hashimoto disease | | Controls |
| --- | --- | --- | --- | --- | --- | --- | --- | --- |
|  | | N=289 | | N=265 | | N=229 | | N=1058 |
| Comparison |  | N(%) | OR(95%CI) | N(%) | OR(95%CI) | N(%) | OR(95%CI) | N(%) |
| +49 A/G G vs. A | | 274(94.8) | **2.16(1.24-3.77)** | 246(92.8) | 1.53(0.92-2.54) | 214(93.4) | 1.69(0.97-2.95) | 946(89.4) |
| CT60 G vs. A | | 286(99.0) | **4.33(1.34-14.04)** | 258(97.4) | 1.68(0.75-3.75) | 222(96.9) | 1.44(0.64-3.24) | 1012(95.7) |
| GG vs. AG^a^ | | 274(95.8) | 1.64(0.88-3.08) | 246(95.3) | 1.48(0.79-2.77) | 214(96.4) | 1.93(0.91-4.07) | 944(93.3) |
| GA vs. AA^a^ | | 0(0.0) | 2.54(0.10-63.99) | 0(0.0) | 1.19(0.05-27.24) | 0(0.0) | 1.19(0.05-27.24) | 2(4.3) |
| GG vs. GA^b^ | | 274(100.0) | 1.45(0.07-30.36) | 246(100.0) | 1.30(0.06-27.27) | 214(100.0) | 1.14(0.05-23.74) | 944(99.8) |
| AG vs. AA^b^ | | 12(80.0) | 2.59(0.69-9.70) | 12(63.2) | 1.11(0.41-3.03) | 8(53.3) | 0.74(0.25-2.18) | 68(60.7) |
| GA vs. AG^c^ | | 0(0.0) | 1.10(0.05-24.22) | 0(0.0) | 1.10(0.05-24.22) | 0(0.0) | 1.61(0.07-36.46) | 2(2.9) |
| GG vs. AA^d^ | | 274(98.9) | 4.26(1.31-13.82) | 246(97.2) | 1.64(0.73-3.68) | 214(96.8) | 1.42(0.63-3.21) | 944(95.5) |
| Association of +49A/G and CT60 | | **153.72*(7.52-3140.40)** | | **295.80*(15.98-5476.93)** | | **378.53*(19.94-7186.63)** | | **305.41*(72.48-1286.85)** |

Significant values are in bold (*P_C_* <0.05). * *Pc* <0.01.

^a^ The two comparisons detect the association between carrier G of +49A/G and AITD in the presence or absence of carrier G of CT60.

^b^ The two comparisons detect the association between carrier G of CT60 and AITD in the presence or absence of carrier G of +49A/G.

^c^ This test detects difference between carrier G of +49A/G and CT60 association with AITD.

^d^ This test detects the association between combined carrier +49G-carrier CT60G and AITD.
